# Involvement of RAMP1/p38MAPK signaling pathway in osteoblast differentiation in response to mechanical stimulation: a preliminary study

**DOI:** 10.1186/s13018-024-04805-w

**Published:** 2024-06-02

**Authors:** Thunwa Binlateh, Chidchanok Leethanakul, Peungchaleoy Thammanichanon

**Affiliations:** 1 School of Pharmacy, Walailak University, Nakhon Si Thammarat, Thailand; 2 Orthodontic Section, Department of Preventive Dentistry, Faculty of Dentistry, Prince of Songkla University, Songkhla, Thailand; 3 Institute of Dentistry, Suranaree University of Technology, Nakhon Ratchasima, Thailand; 4 Oral Health Center, Suranaree University of Technology Hospital, Suranaree University of Technology, Nakhon Ratchasima, Thailand

**Keywords:** CGRP, Mechanical loading, Osteoblast, RAMP1

## Abstract

**Objective:**

The present study aimed to investigate the underlying mechanism of mechanical stimulation in regulating osteogenic differentiation.

**Materials and methods:**

Osteoblasts were exposed to compressive force (0–4 g/cm^2^) for 1–3 days or CGRP for 1 or 3 days. Expression of receptor activity modifying protein 1 (RAMP1), the transcription factor RUNX2, osteocalcin, p38 and p-p38 were analyzed by western blotting. Calcium mineralization was analyzed by alizarin red straining.

**Results:**

Using compressive force treatments, low magnitudes (1 and 2 g/cm^2^) of compressive force for 24 h promoted osteoblast differentiation and mineral deposition whereas higher magnitudes (3 and 4 g/cm^2^) did not produce osteogenic effect. Through western blot assay, we observed that the receptor activity-modifying protein 1 (RAMP1) expression was upregulated, and p38 mitogen-activated protein kinase (MAPK) was phosphorylated during low magnitudes compressive force-promoted osteoblast differentiation. Further investigation of a calcitonin gene-related peptide (CGRP) peptide incubation, a ligand for RAMP1, showed that CGRP at concentration of 25 and 50 ng/ml could increase expression levels of RUNX2 and osteocalcin, and percentage of mineralization, suggesting its osteogenic potential. In addition, with the same conditions, CGRP also significantly upregulated RAMP1 and phosphorylated p38 expression levels. Also, the combination of compressive forces (1 and 2 g/cm^2^) with 50 ng/ml CGRP trended to increase RAMP1 expression, p38 activity, and osteogenic marker RUNX2 levels, as well as percentage of mineralization compared to compressive force alone. This suggest that RAMP1 possibly acts as an upstream regulator of p38 signaling during osteogenic differentiation.

**Conclusion:**

These findings suggest that CGRP-RAMP1/p38MAPK signaling implicates in osteoblast differentiation in response to optimal magnitude of compressive force. This study helps to define the underlying mechanism of compressive stimulation and may also enhance the application of compressive stimulation or CGRP peptide as an alternative approach for accelerating tooth movement in orthodontic treatment.

## Introduction

The basis of orthodontic tooth movement is the physiologic alveolar bone adaptation to the mechanical strains. When mechanical force is applied to the teeth, osteoblasts within the tissue detect and translate the mechanical signals to intracellular biological responses [1]. Mechanical stimulation enhances alkaline phosphatase activity, calcium deposition, and expression levels of osteoblast markers RUNX2 and osteocalcin, thereby regulating osteogenic differentiation to achieve the movement of orthodontic teeth [2]. In clinical reserach, mechanical stimulation can reduce root resorption [3] and increase post-treatment bone density in orthodontic treatment [4]. Although many publications demonstrated the effect of mechanical stimulation on osteogenic differentiation, its underlying mechanism is poorly defined.

Emerging evidence has proposed the involvement of neuropeptides in regulating bone formation, repair and regeneration. Among neuropeptides identified in the bone, calcitonin gene-related peptide (CGRP) has been considered as a key neuropeptide implicated in bone metabolism [5]. CGRP, a member of the calcitonin protein family, is a 37-amino acid peptide that is released from sensory nerve ending [6]. CGRP can promote osteogenesis, inhibit bone resorption and promote vascular growth [7]. With these functions, CGRP requires its related regulatory protein, a receptor activity modifying protein 1 (RAMP1). RAMP1 belongs to a family of proteins with single transmembrane protein, including three different members: RAMP1, RAMP2 and RAMP3. RAMP1 confers ligand specificity for CGRP to regulate osteogenic factors and promote osteoblast anabolism, while RAMP2 and RAMP3 are responsible for binding with adrenomedullin [8]. 

A growing body of evidence has shown that p38 mitogen-activated protein kinase (MAPK) regulates several aspects of bone development, including osteoblast proliferation and differentiation, extracellular matrix deposition, and mineralization [9]. The p38MAPK signaling pathway can be activated in response to various extracellular signals. Previous in vitro studies revealed that different osteogenic ligands such as BMP2, TGF-ß, and Wnt proteins could stimulate p38MAPK to promote osteogenic differentiation and organic mineral deposition [10, 11]. In addition, recent investigation also demonstrated that CGRP treatment induces osteoblast differentiation of bone marrow mesenchymal stem cells via p38MAPK activation [12]. Although many studies have reported the role of p38MAPK on osteogenic effect in response to various osteogenic ligands, it is still a lack of evidence revealing the involvement of this signaling pathway in mechanical stimulation-induced osteogenesis.

Based on these, the core issue of this work was therefore to investigate the mechanism underlying osteogenic differentiation in response to mechanical force. Here, we found that optimal magnitude of compressive loading (1–2 g/cm^2^) exerts an osteogenic effect. More importantly, osteogenic potential of compressive force involves RAMP1/p38MAPK activation. This study not only helps to define the underlying mechanism of compressive stimulation, but may also enhance the use of compressive force and CGRP peptide as a promising alternative approach for accelerating orthodontic tooth movement.

## Materials and methods

### Isolation of osteoblasts from alveolar bone and cell culture

**Fig. 1:**
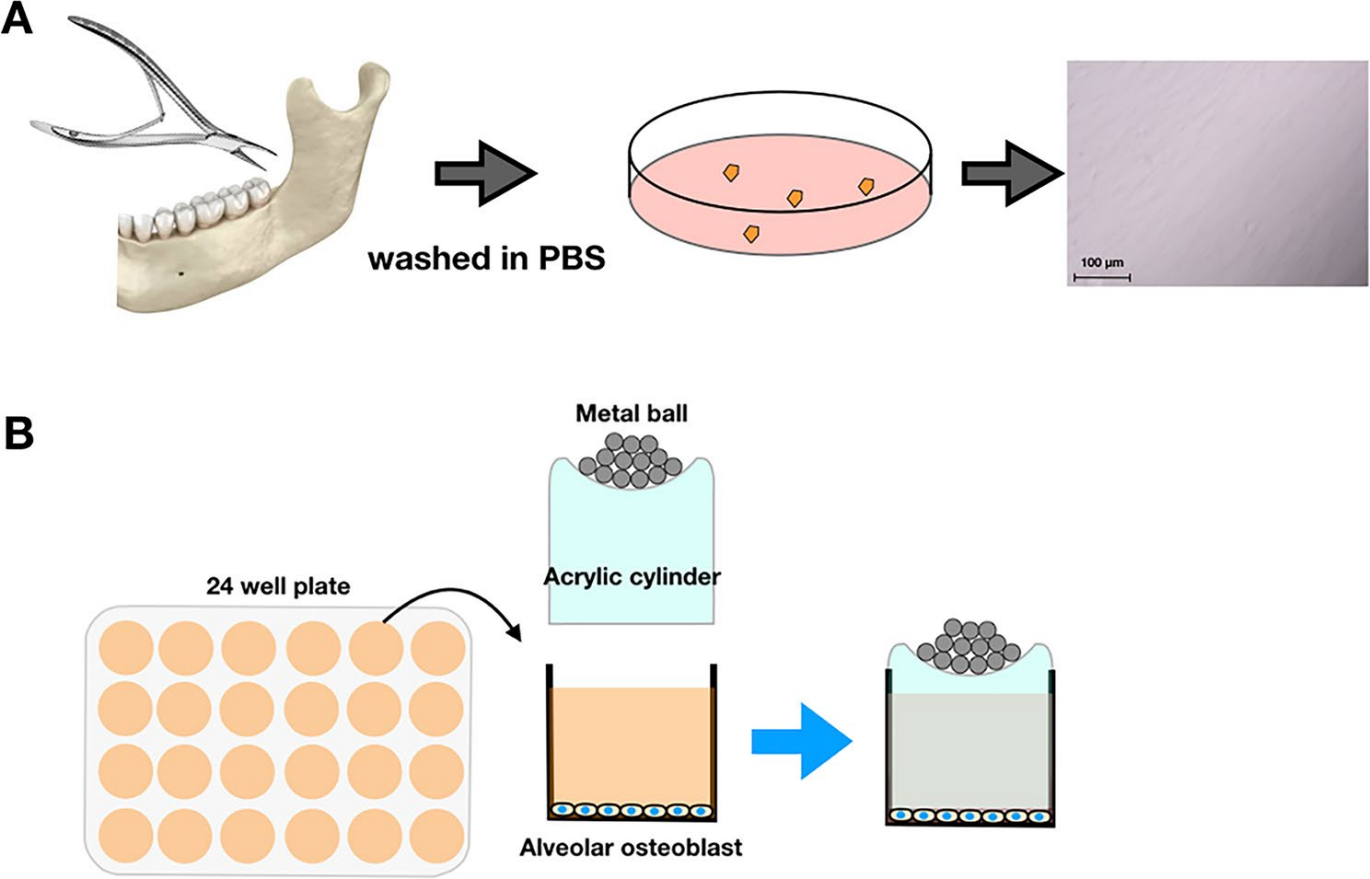
**A** A diagram presenting isolation of primary alveolar osteoblast cells. **B** Compressive force application model, performed in a 24-well plate

Jaw bone samples were obtained from five healthy individuals aged 17–25-years-old during orthognathic surgery of the mandible after obtaining informed consent and the approval of the Ethics Committee Board of Prince of Songkla University, Songkhla, Thailand (EC6609-046). Primary osteoblast cells were isolated by explantation culture, as described in previous studies [13]. Briefly shown in Fig. 1A, the alveolar bone pieces were thoroughly washed in phosphate buffered saline (PBS) to remove any soft tissue and periosteum and vortexed. The specimens were transferred to culture dishes (Corning, Glendale, AZ, USA) and then incubated in α-Modified minimal essential medium (αMEM; Gibco BRL, Grand Island, NY, USA) containing 10% fetal bovine serum (FBS; Gibco BRL), 1% penicillin/streptomycin (Gibco BRL), and 1% fungizone solution (Gibco BRL) at 5% CO_2_ and 37 °C. Cells from the third to fourth passages were used for the experiments.

For the bone differentiation, the cells were cultured in the α-MEM supplemented with 10% FBS, 50 mM ascorbic acid, 100 mM dexamethasone and 10 mM β-glycerophosphate for 7–21 days.

### Application of compressive force

The protocol of mechanical force application in this study was modified from previously described method [14, 15]. To stimulate compressive force, osteoblasts in 24-well plates were subjected to continuous compressive force varying from 1.0 to 4.0 g/cm^2^ using an acrylic cylinder containing a metal ball as shown in Fig. 1B. Control cells were not exposed to compressive force. All experiments were performed in triplicate for each sample.

### CGRP peptide treatment

CGRP peptide treatment was adapted from the protocol of previous studies [16]. To synchronize the osteoblasts at the G0 phase and study the effect of CGRP treatment on bone formation, the osteoblasts were incubated in serum-free DMEM culture media in 24-well plates for 24 h at 37 °C. The synchronized cells were then divided into five groups and exposed to different conditions: Group 1 served as the negative control, and were cultured in DMEM. Group 2 were treated with DMEM containing 25 ng/mL CGRP. Group 3 were treated with DMEM containing 50 ng/mL CGRP. Group 4 were treated with DMEM containing 100 ng/mL CGRP. Lastly, Group 5 were treated with DMEM containing 200 ng/mL CGRP.

### MTT assay of cell viability

The MTT assay was utilized to assess the viability and proliferation of the osteoblast cells over a period of up to 3 days. MTT reagent (Sigma-Aldrich, Inc., St. Louis, MO, USA) was added to each sample, and the cells were incubated at 37 °C in a humidified atmosphere with 5% CO_2_ for 3 h to allow the formation of MTT formazan. After incubation, the cells were washed with PBS and the formazan was dissolved in dimethyl sulfoxide (DMSO; Sigma-Aldrich, Inc.). The absorbance of each solution was measured at a wavelength of 570 nm using a microplate reader (Bio-Rad, Hercules, CA, USA) in triplicate. The viability and proliferation of osteoblast cells was expressed as relative change in comparison to the control  [17]. 

### Alizarin red staining

Calcium deposits of differentiated osteoblasts were identified by Alizarin red staining. After 7–21 days of culture with differentiation medium, cells were fixed in 4% paraformaldehyde (Sigma-Aldrich) for 20 min, followed by three washes with phosphate-buffered saline (PBS). The fixed cells were stained for 5 min with alizarin red (Sigma-Aldrich) and viewed using Zen software version 2.6 blue edition (Carl Zeiss, Oberkochen, Germany).

### Western blotting

The expression of RAMP1, RUNX2, osteocalcin, p38 and p-p38 in osteoblasts were evaluated by western blot analysis. After the experiments, the cells were lysed using lysis buffer (Cell Signaling Technology, Inc., Danvers, MA, USA). The protein concentration was quantified using the BCA protein assay kit (Pierce™, Waltham, MA, USA). Each protein sample was separated by 10% SDS-PAGE and transfered onto PVDF membranes. Then, the blots were blocked with 5% skimmed milk solution and 3% BSA solution (for phosphor proteins) for 60 min at room temperature. The blots were incubated with polyclonal rabbit anti-RAMP1 (1:500; Abcam, Cambridge, MA, USA), polyclonal rabbit anti-RUNX2 (1:500; Abcam), polyclonal rabbit anti- osteocalcin (1:500; Abcam), polyclonal rabbit anti-p38 (1:1000; Cell Signaling, Denvers, MA, USA) or polyclonal rabbit anti-p-p38 (1:1000; Cell Signaling) overnight at 4 °C. Then, the membranes were washed with Tris-buffered saline for 5 min three times and incubated with HRP-conjugated goat anti-rabbit IgG (1:5,000; Invitrogen, Carlsbad, CA, USA) or goat anti-mouse IgG (1:3,000; Invitrogen) for 60 min at room temperature. Protein band densities were detected and analyzed by a ChemiDoc XRS System (Bio-Rad, Hercules, CA, USA).

### Statistical analysis

The data are expressed as the mean ± standard deviation of measurements obtained from osteoblasts isolated from five independent donors. Each measurement was performed in triplicate. One-way analysis of variance (ANOVA) was conducted to compare the mean values between different groups in cell viability, percentage of mineralization, and protein expression of RAMP1, RUNX2, osteocalcin, p38 and p-p38 in alveolar osteoblasts. Statistical significance was determined as *P* < 0.05.

## Results

### Effect of compressive forces on osteoblast differentiation

**Fig. 2:**
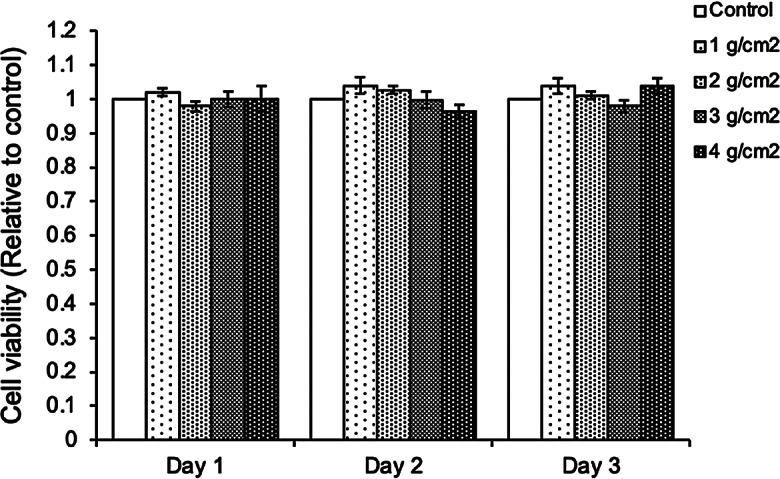
Viability of cells analyzed by MTT assay after being treated with 1–4 g/cm^2^ for 1–3 days. Data are expressed as mean ± SD (*n* = 5 for each group in triplicate); *p < 0.05 and **p < 0.01 compared with the control group, one-way ANOVA

**Fig. 3:**
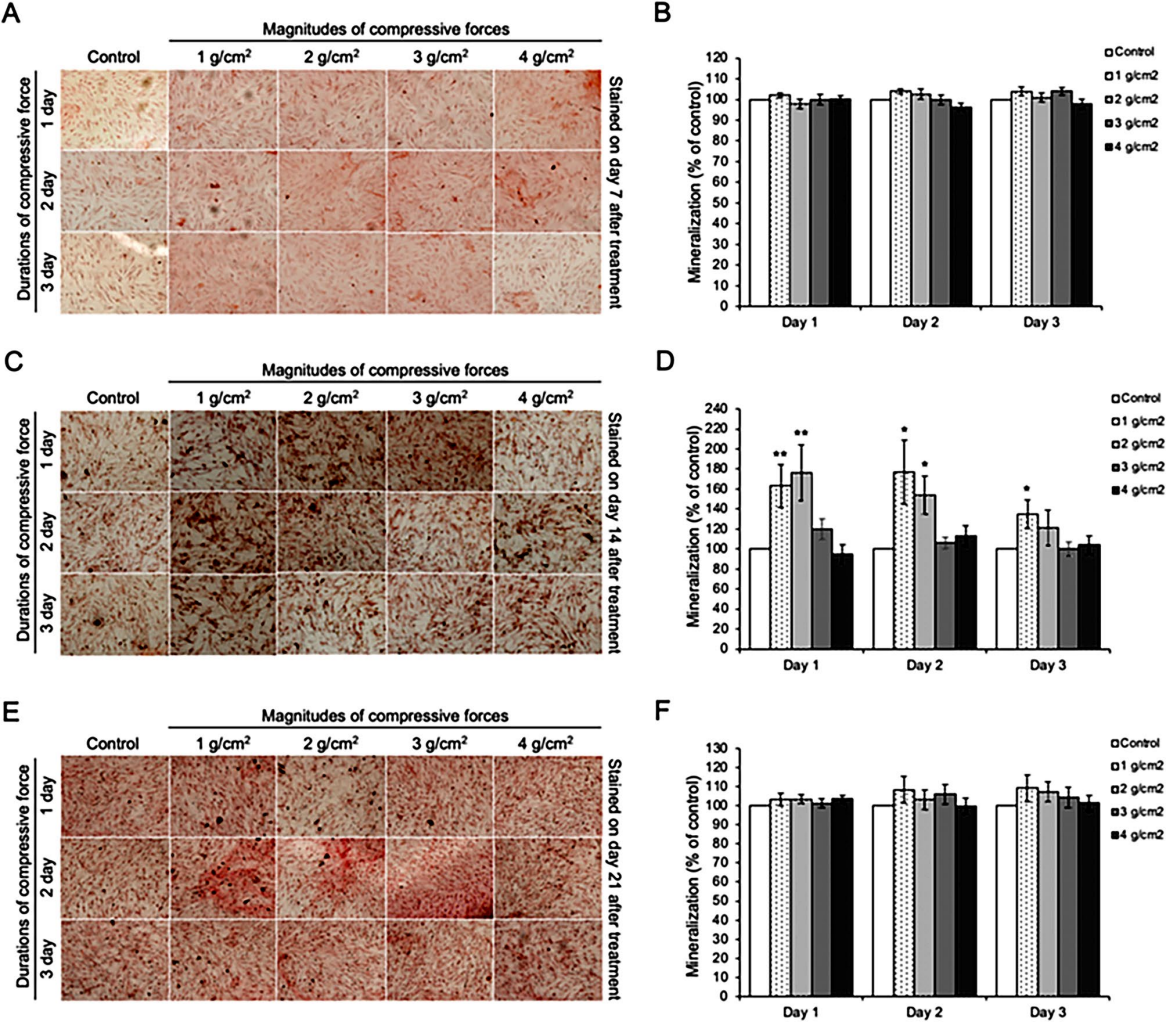
Effect of different magnitudes and durations of compressive force on osteoblast differentiation. (**A**, **C**, **E**) Alizarin red staining, and (**B**, **D**, **F**) Percentage of mineralization relative to control cells after being treated with 1–4 g/cm^2^ for 1–3 days on day 7, 14 and 21. Data are expressed as mean ± SD (*n* = 5 for each group in triplicate); *p < 0.05 and **p < 0.01 compared with the control group, one-way ANOVA

Initially, the viability of alveolar pre-osteoblasts after being treated with 1–4 g/cm^2^ compressive force for 1–3 days was determined. As shown in Fig. 2, there were no detectable changes in cell viability in any treatment groups at any time points. We next investigated the effects of compressive force on osteoblast differentiation. The alveolar pre-osteoblasts were exposed to the 1–4 g/cm^2^ magnitudes of compressive force for 1, 2, or 3 days in bone differentiation medium, and the percentage of mineralization was analyzed by Alizarin red staining at day 7, 14, and 21 after treatment. It was noticed that exposure to 1 and 2 g/cm^2^ compressive force for 1 and 2 days significantly increased the percentage of mineralization on day 14 compared to control cells; however, no significant differences were observed on days 7 and 21. Higher levels (3 and 4 g/cm^2^) of compressive force did not significantly increase the percentage of mineralization compared to control cells (Fig. 3A–F).

**Fig. 4:**
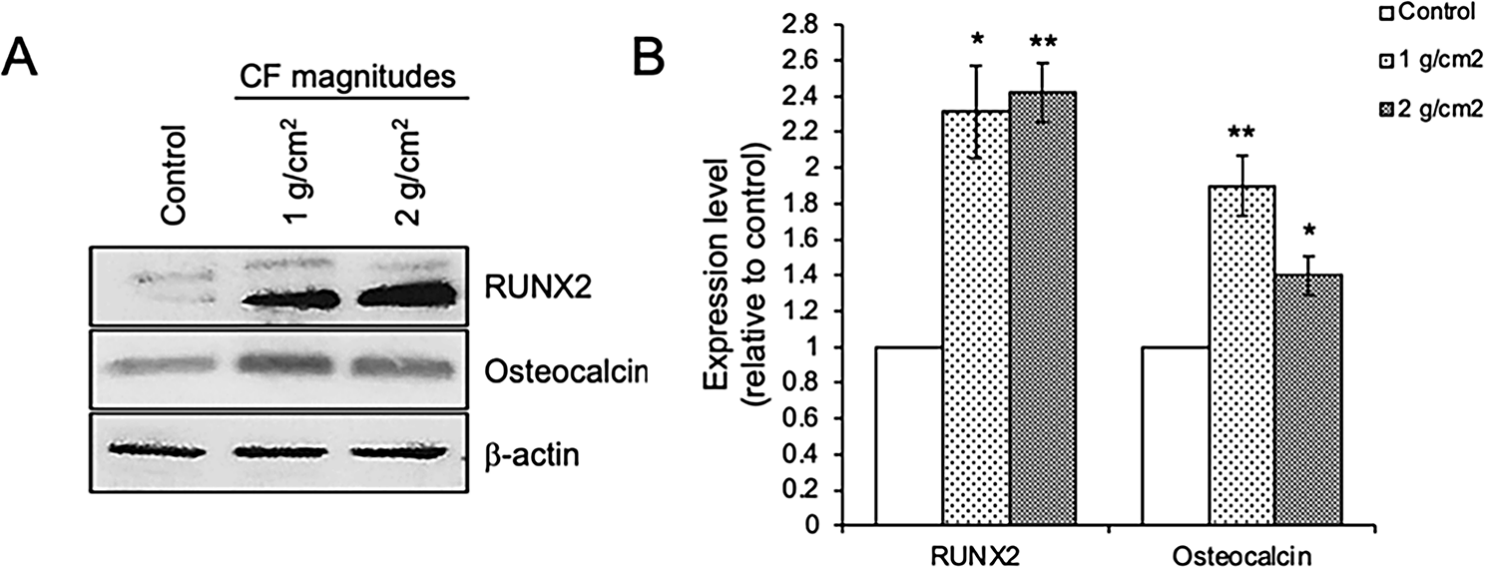
Effect of compressive forces on osteogenic marker expression. (**A**) Immunoblotting analysis of RUNX2 and osteocalcin, (**B**) Quantitative expression levels of RUNX2 and osteocalcin after being treated with 1 and 2 g/cm^2^ for 1 day. Data are expressed as mean ± SD; (*n* = 5 for each group in triplicate); *p < 0.05 and **p < 0.01 compared with the control group, one-way ANOVA

To confirm the differentiation of alveolar osteoblast after being treated with 1 and 2 g/cm^2^ compressive forces, the expression levels of osteoblast differentiation markers, RUNX2 and osteocalcin, were determined at day 7 after treatment. Western blotting results showed a significant upregulation of RUNX2 and osteocalcin expressions in both magnitudes of compressive force treatment compared to control cells (Fig. 4A and B). These results indicate that 1 and 2 g/cm^2^ compressive forces accelerate alveolar osteoblast differentiation.

### Treatment with compressive forces upregulates RAMP1 and p38

**Fig. 5:**
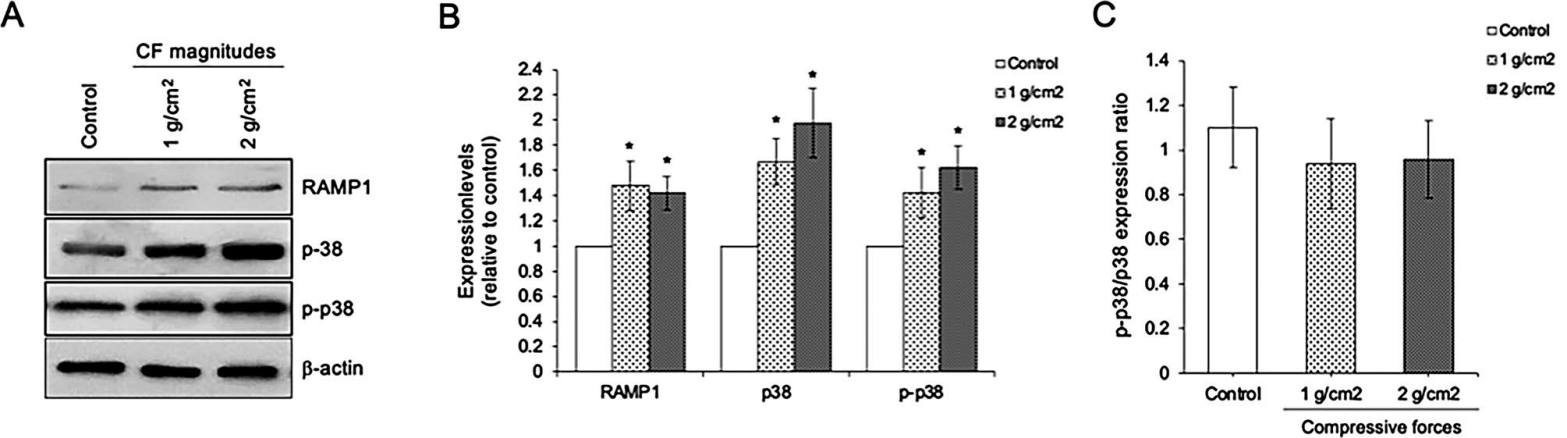
Compressive force upregulates RAMP1 and activates p38. (**A**) Immunoblotting analysis of RAMP1 p38, and p-p38, (**B**) Quantitative expression levels of RAMP1 p38, and p-p38, and (**C**) Expression ratio of p-p38/p38 after being treated with 1 and 2 g/cm^2^ for 1 day. Data are expressed as mean ± SD(*n* = 5 for each group in triplicate); *p < 0.05 and **p < 0.01 compared with the control group, one-way ANOVA

RAMP1 is a receptor component of CGRP which has been reported to involve osteoblast formation via p38 MAPK signaling. To determine whether RAMP1 and p38 complicate in compressive-accelerated alveolar osteoblast, the expression of RAMP1, p38 and p-p38 was investigated. It was shown a significant upregulation of RAMP1, p38 and p-p38 expression levels after being treated with 1 and 2 g/cm^2^ compressive forces compared to control cells (Fig. 5A and B). However, the expression ratio of p-p38/p38 in both experimental group were no significant difference compared to control cells (Fig. 5C).

### CGRP receptor component RAMP1 involves alveolar osteoblast differentiation

**Fig. 6:**
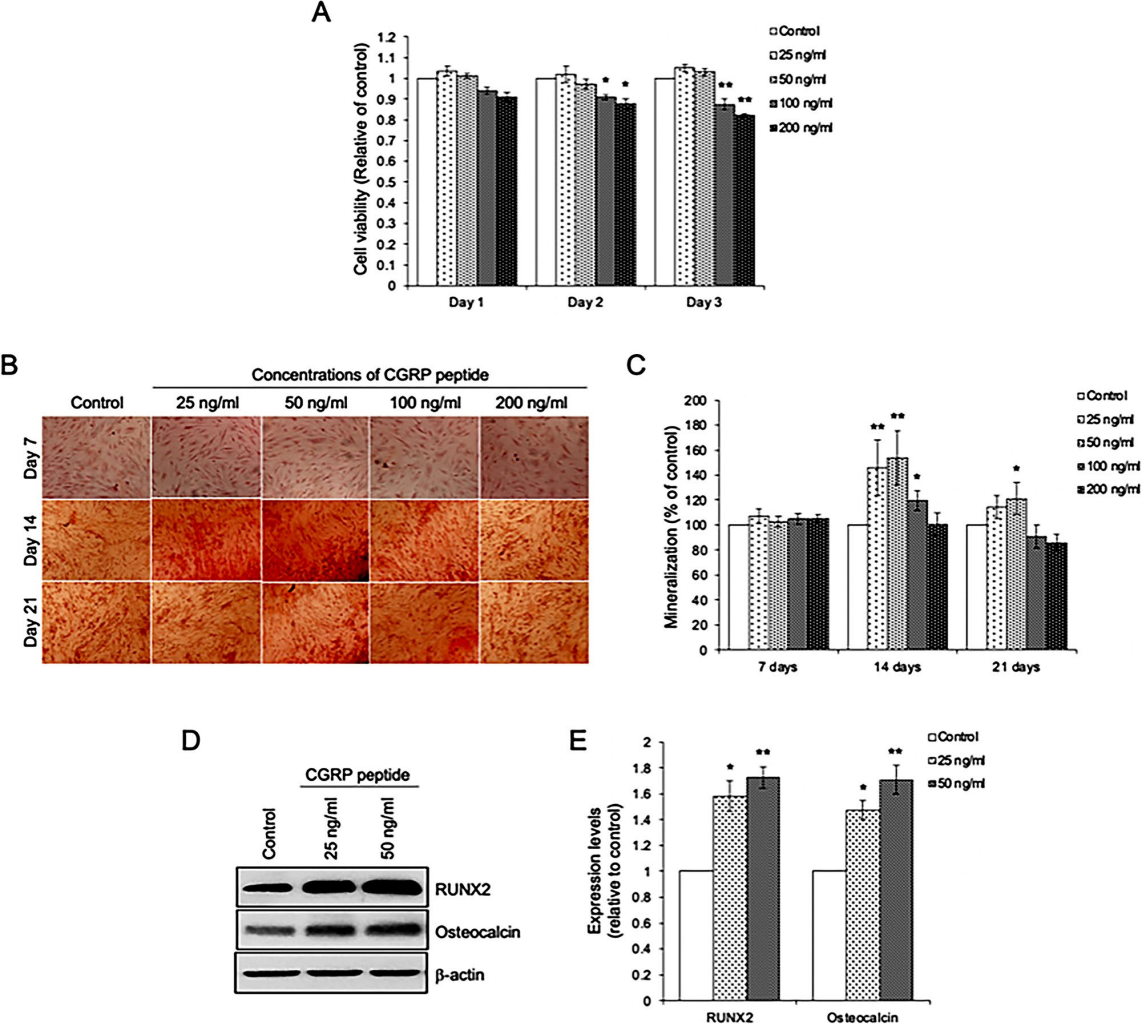
CGRP peptide promotes osteoblast differentiation. (**A**) Viability of cells after being incubated with CGRP peptide at concentrations of 25–200 ng/ml for 1–3 days. (**B**) Alizarin red staining, and (**C**) Percentage of mineralization relative to control of cells after being treated with 25–200 ng/ml for 24 h. (**D**) Immunoblotting analysis of RUNX2 and osteocalcin, and (*E*) Quantitative expression levels of RUNX2 and osteocalcin after being treated with 25 and 50 ng/ml of CGRP peptide for 1 day. Data are expressed as mean ± SD(*n* = 5 for each group in triplicate); *p < 0.05 and **p < 0.01 compared with the control group, one-way ANOVA

To verify the involvement of RAMP1 on osteogenic differentiation, alveolar osteoblasts were incubated with CGRP peptide. Firstly, the viability of alveolar osteoblasts after being exposed to 25–200 ng/ml was assessed. While the lower concentrations (25 and 50 ng/ml) of CGRP peptide did not cause a significant reduction in cell viability, the higher doses (100 and 200 ng/ml) significantly decreased the viability of cells (Fig. 6A). The percentage of mineralization was further analyzed, and found that 25 and 50 ng/ml of CGRP peptide treatment significantly increased the percentage of mineralization at day 14 after treatment compared to control cells (Fig. 6B and C). Consistently, the expression levels of RUNX2 and osteocalcin at 14 days after being incubated with 25 and 50 ng/ml CGPR peptide were significantly increased (Fig. 6D and E). These results suggest that CGRP/RAMP1 involves alveolar osteoblast differentiation.

### CGRP/RAMP1 regulates osteoblast differentiation through p38 MAPK

**Fig. 7:**
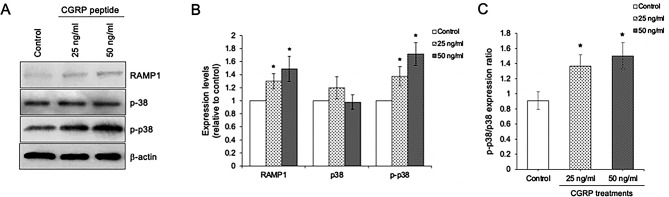
CGRP peptides upregulates RAMP1 and activates p38. (**A**) Immunoblotting analysis of RAMP1 p38, and p-p38, and (**B**) Quantitative expression levels of RAMP1 p38, and p-p38, and (**C**) Expression ratio of p-p38/p38 after being treated with 25 and 50 ng/ml of CGRP peptide for 1 day. Data are expressed as mean ± SD(*n* = 5 for each group in triplicate); *p < 0.05 and **p < 0.01 compared with the control group, one-way ANOVA

Recent study has reported that CGRP/RAMP1 exerts osteogenic differentiation of mesenchymal stem cells through p38 MAPK signaling [18]. We thus investigated the expression of RAMP1, p38 and p-p38 in the CGRP peptide-treated osteoblasts. The results exhibited that CGRP peptide significantly increased the expression levels of RAMP1, p-p38, and p-p38/p38 expression ratio compared to control cells, while p38 expression was not affected (Fig. 7A–C). These data indicate that CGRP/RAMP1 regulates osteoblast differentiation through p38 signaling.

### RAMP1 possibly acts as an upstream regulator of p38 in response to osteoblast differentiation induced by compressive force

**Fig. 8:**
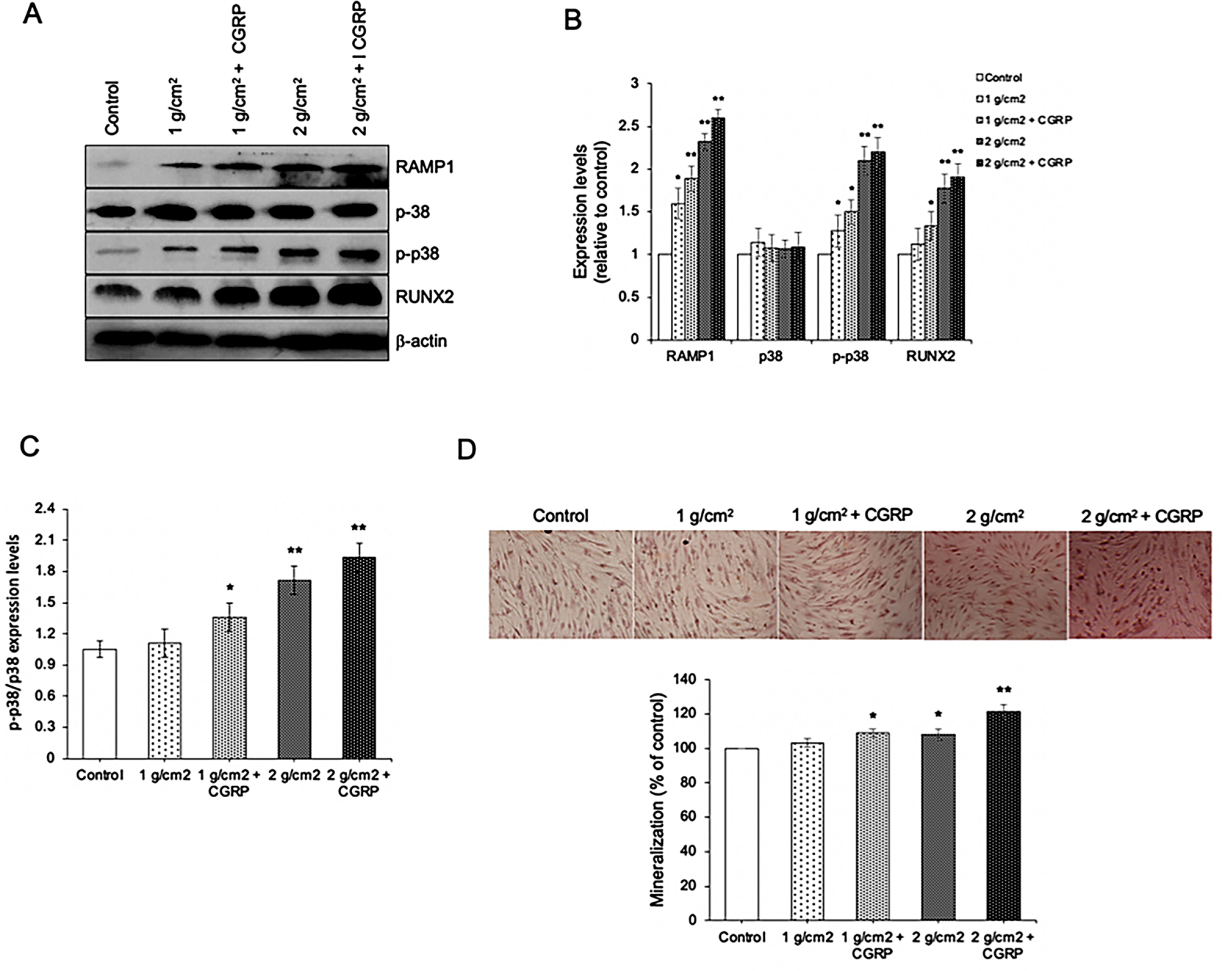
RAMP1 possibly acts as an upstream regulator of p38 in response to osteoblast differentiation induced by compressive force. (A) Immunoblotting analysis of RAMP1, p38, p-p38, and RUNX2, (B) Quantitative expression levels of RAMP1, p38, p-p38, and RUNX2, and (C) Expression ratio of p-p38/p38 after being exposed to compressive forces (1–2 g/cm^2^) and compressive forces combined with 50 ng/ml CGRP. (D) Alizarin red staining of cells after being exposed to compressive forces (1–2 g/cm^2^) and compressive forces combined with 50 ng/ml CGRP. Data are expressed as mean ± SD(*n* = 5 for each group in triplicate); *p < 0.05 and **p < 0.01 compared with the control group, one-way ANOVA

In order to examine whether RAMP1 or p38 acts as an upstream regulator of osteoblast differentiation after compressive force stimulation, the expression levels of RAMP1, p38, p-p38, and RUNX2 and percentage of mineralization were determined after being exposed to compressive forces (1–2 g/cm^2^) combined with 50 ng/ml CGRP and compressive forces only. The results showed that treatments with compressive forces and combination of compressive forces with CGRP significantly upregulated RAMP1, p-p38, and RUNX2 compared to control. In comparison between compressive forces and combination groups, it was found that the combination groups trended to upregulate all protein expression levels, although they were not statistical difference (Fig. 8A-C). Consistently, alizarin red data showed that both compressive forces only and combination groups significantly increased the percentage of mineralization. Furthermore, the percentage of mineralization also trended to higher in the combination treatment (Fig. 8D). These data imply that RAMP1 may act as the upstream regulator of p38 during compressive force-induced osteoblast differentiation.

## Dis**cussion**

Despite many publications convincingly demonstrated the potential of mechanical force on osteogenic differentiation, it is still undefined how mechanical stimulation regulates osteogenic differentiation. The present study demonstrated that treatment with low magnitudes of compressive force (1 and 2 g/cm^2^), but not high magnitudes (3 and 4 g/cm^2^), for 24 h promoted osteogenic differentiation and mineral deposition. The CGRP component receptor RAMP1 and p38MAPK signaling pathways participated in osteogenic differentiation in response to compressive force. In addition, incubating with CGRP peptide, a specific ligand of RAMP1, also induced differentiation of osteoblasts with increased phosphorylated levels of p38. These data implicate the RAMP1/p38MAPK signaling pathway in the osteogenic differentiation.

Mechanical stimulation, especially compressive force, has been extensively demonstrated to regulate osteogenic differentiation [19, 20]. The present study showed that compressive load promotes osteoblast differentiation when the optimal magnitudes of 1 and 2 g/cm^2^ are applied, while higher magnitudes (3 and 4 g/cm^2^) of compressive force do not stimulate osteoblastic differentiation. These findings are consistent with previous research that showed the optimal magnitude of compressive force upregulated the expression of factors and markers involved in osteoblast differentiation. For instance, Shionome et al. [21] revealed that 1 and 2 g/cm^2^ were the optimal magnitudes of compressive force that upregulated RUNX2 and BMP2. In addition, Chen et al. [22] demonstrated that an optimal magnitude of compressive force (0.33–0.5 MPa) increased alkaline phosphatase activity and RUNX2 expression in a 3D scaffold bone model. In contrast, higher-magnitude compressive force (1–1.7 MPa) induced expression of pro-inflammatory factors like IL-6 and COX-2 and downregulated osteoprotegerin in osteoblast [23]. Furthermore, we also examined the effects of different durations of compressive load treatment. Exposure to compressive force for 1 and 2 days effectively increased Alizarin red staining of mineral nodules and upregulated RUNX2 expression at 14 days after treatment. It has previously been reported that the response of osteogenic cells to compressive load depends on the magnitude and duration of force. High magnitude (1 MPa) treatment for 6 h dramatically increased RUNX2 expression in a 3D scaffold bone model compared to 8 h treatment [24]. In another bone model, a high strain magnitude decreased *RUNX2* mRNA expression at 24 and 48 h in stromal ST2 cells. These indicate that both the magnitude and duration of compressive load affect the osteogenic response.

RAMP1 is part of the CGRP receptor complex which is responsible for CGRP binding. CGRP is one of neuropeptide identified in bone tissue, and has been considered to play a critical role in the skeletal development and bone metabolism [25]. Our study preliminarily investigated the change in expression of RAMP1 after being compressed with low magnitudes (1 and 2 g/cm^2^) for 24 h, and found that the levels of RAMP1 were promoted. In addition, treatment with CGRP peptide not only upregulated RAMP1 expression, but also increased percentage of mineralization, and expression of osteogenic markers. These observations were in parallel with the previous work that CGRP incubation promote differentiation capacity of bone mesenchymal stem cells toward osteoblasts as accompanied by increased mRNA and protein expression of collagen type I, osteopontin, and RUNX2, as well as calcified nodules [26]. These findings suggest the role of CGRP-RAMP1 on osteoblast differentiation, and the participation of RAMP1 in the differentiation of osteoblast in response to compressive force.

p38MAPK signaling pathway is an intracellular cascade implicated in a wide variety of physiological events such as cell survival, proliferation, differentiation, and others [27]. While some studies have reported the positive potential of p38MAPK in regulating osteogenic differentiation and bone formation [28, 29], other researched has revealed its negative effects by causing osteoclast maturation and bone loss [12]. The present findings showed that p38MAPK signaling is activated during compressive force- and CGRP peptide-induced osteoblast differentiation, suggesting its role on regulating osteoblast differentiation. Consistent to the previous study, CGRP treatment led to increase differentiation capacity into osteoblasts, as indicated by the increased RUNX2 level and mineralization, through p38MAPK and its downstream cascade ERK1/2 stimulation [30]. Several studies, using both conditional knockout and pharmacological inhibition of p38MAPK signaling have suggested that RUNX2 is a target of p38 kinase activity, boosting its transcriptional potential [9, 28, 31, 32]. RUNX2 is responsible for activating osteoblast differentiation markers such as alkaline phosphatase, osteocalcin and osteopontin, as well as mineral deposition [33]. These literatures, together with the recent findings indicated that compressive force and CGRP peptide regulates osteogenic differentiation via p38MAPK activation.

**Fig. 9:**
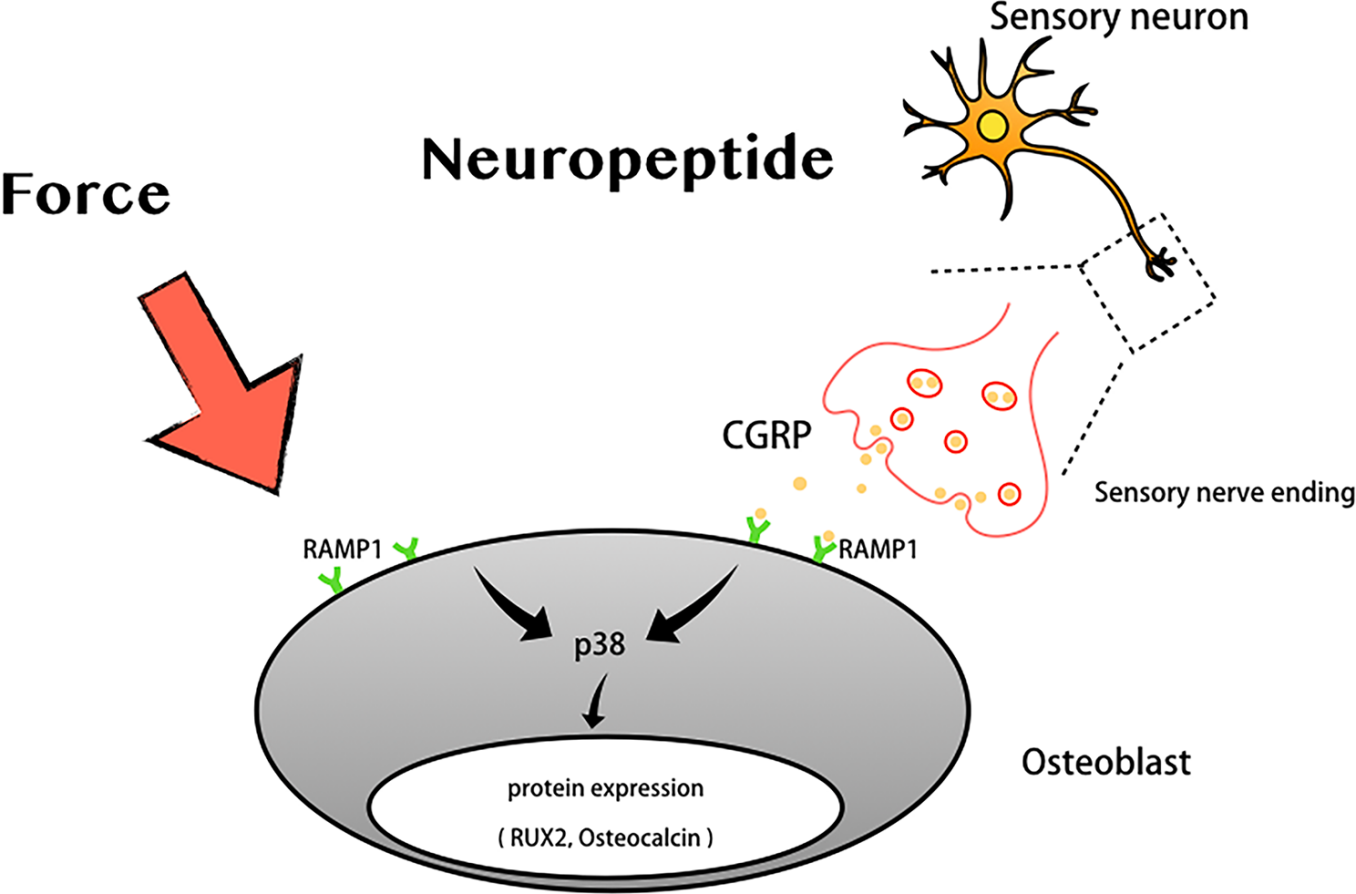
Schematic diagram representing the mechanism underlying compressive force promoted osteoblast differentiation

In summary, the present study found that application of the optimal magnitude of compressive loading (1–2 g/cm^2^) exerts an osteogenic effect through RAMP1/p38MAPK signaling activation, as shown in Fig. 9. Collectively, this study helps to define the underlying mechanism of compressive force, and also enhance the translation to clinical use of compressive force and CGRP peptide as the futuristic promising method for the accelerated orthodontic tooth movement.

## Data Availability

The data that support the findings of this study are available from the corresponding author upon reasonable request.
