# Correction to: Involvement of RAMP1/p38MAPK signaling pathway in osteoblast differentiation in response to mechanical stimulation: a preliminary study

**DOI:** 10.1186/s13018-024-04872-z

**Published:** 2024-07-09

**Authors:** Thunwa Binlateh, Chidchanok Leethanakul, Peungchaleoy Thammanichanon

**Affiliations:** 1 School of Pharmacy, Walailak University, Nakhon Si Thammarat, Thailand; 2 Orthodontic Section, Department of Preventive Dentistry, Faculty of Dentistry, Prince of Songkla University, Songkhla, Thailand; 3 Institute of Dentistry, Suranaree University of Technology, Nakhon Ratchasima, Thailand; 4 Oral Health Center, Suranaree University of Technology Hospital, Suranaree University of Technology, Nakhon Ratchasima, Thailand

**Correction to: Journal of Orthopaedic Surgery and Research (2024) 19:330**

10.1186/s13018-024-04805-w

The ordering numbers of in-text citation in the above publication are not ordered as an in-text citation number 13 was skipped. The in-text citation number 13 should be instead of number 14 in the part of “Isolation of osteoblasts form alveolar bone and cell culture” of Materials and methods. Then, the in-text citation number 14 should be instead of number 15, and so on.

The original article has been corrected.

